# Origin and evolution of mitochondrial inner membrane composition

**DOI:** 10.1242/jcs.263780

**Published:** 2025-04-23

**Authors:** Kailash Venkatraman, Nicolas-Frédéric Lipp, Itay Budin

**Affiliations:** Department of Chemistry and Biochemistry, University of California San Diego, La Jolla, CA 92093, USA

**Keywords:** Mitochondria, Cristae, Cardiolipin, Evolution, Curvature, Phospholipids

## Abstract

Unique membrane architectures and lipid building blocks underlie the metabolic and non-metabolic functions of mitochondria. During eukaryogenesis, mitochondria likely arose from an alphaproteobacterial symbiont of an Asgard archaea-related host cell. Subsequently, mitochondria evolved inner membrane folds known as cristae alongside a specialized lipid composition supported by metabolic and transport machinery. Advancements in phylogenetic methods and genomic and metagenomic data have suggested potential origins for cristae-shaping protein complexes, such as the mitochondrial contact site and cristae-organizing system (MICOS). MICOS protein homologs function in the formation of cristae-like intracytoplasmic membranes (ICMs) in diverse extant alphaproteobacteria. The machinery responsible for synthesizing key mitochondrial phospholipids – which cooperate with cristae-shaping proteins to establish inner membrane architecture – could have also evolved from a bacterial ancestor, but its origins have been less explored. In this Review, we examine the current understanding of mitochondrial membrane evolution, highlighting distinctions between prokaryotic and eukaryotic mitochondrial-specific proteins and lipids and their differing roles in shaping cristae and ICM architecture, and propose a model explaining the concurrent specialization of the mitochondrial lipidome and inner membrane structure in eukaryogenesis. We discuss how advancements across a range of disciplines are shedding light on how multiple membrane components co-evolved to support the central functions of eukaryotic mitochondria.

## Teaser

**Summary:** An examination of the morphological similarities between prokaryotic intracytoplasmic membranes and mitochondrial inner membranes, and whether cristae evolution has driven specialization of the mitochondrial lipidome.

## Introduction

Mitochondria are double membrane-bound organelles that are the main sites of bulk ATP synthesis in most eukaryotic cells. The outer mitochondrial membrane (OMM) is highly permeable, allowing for diffusion of metabolites and small molecules into and out of mitochondria (Camara et al., 2017; Helle et al., 2013). In contrast, the inner mitochondrial membrane (IMM) houses bioenergetic complexes of the electron transport chain (ETC) and maintains a proton gradient that drives ATP synthesis (Vercellino and Sazanov, 2022). Additionally, the IMM is organized into highly curved inward folds known as cristae, which increase the membrane area for the ETC (Mannella et al., 2001, 2013). Cristae exhibit significant diversity across eukaryotes – and even between tissues in metazoans – and remodel according to metabolic needs (Cogliati et al., 2016; Pánek et al., 2020). The formation of cristae requires the cooperative action of three main cristae-shaping protein complexes: ATP synthase dimers, which induce membrane bending at cristae tips (Blum et al., 2019; Davies et al., 2011, 2012; Strauss et al., 2008) optic atrophy protein 1 (OPA1), which is a dynamin-related GTPase (Frezza et al., 2006; Patten et al., 2014), and the mitochondrial contact site and cristae-organizing system (MICOS) complex (Harner et al., 2011; Hoppins et al., 2011). OPA1 and the MICOS complex physically interact at animal crista junctions (Glytsou et al., 2016; Hu et al., 2020; Schweppe et al., 2017). Although the protein machinery involved in cristae formation has been extensively characterized, emerging evidence reveals distinct roles for mitochondrial phospholipids (PLs) in orchestrating membrane architecture and organization (Ikon and Ryan, 2017; Joshi et al., 2023; Venkatraman et al., 2023, 2024). Notably, abundant mitochondrial PLs like phosphatidylethanolamine (PE) and cardiolipin (CL), which do not spontaneously form bilayers, are known to interact with cristae-shaping proteins (Aaltonen et al., 2016; Ban et al., 2017; Friedman et al., 2015; Liu and Chan, 2017; Mårtensson et al., 2017; Mehdipour and Hummer, 2016; Mühleip et al., 2019) and might modulate cristae ultrastructure through their intrinsic molecular geometry (Decker and Funai, 2024; Venkatraman et al., 2023, 2024).

The distinctive architecture of highly curved cristae membranes observed in contemporary eukaryotes likely emerged during early mitochondrial evolution. The hypothesis that mitochondria were once free-living bacteria that became eukaryotic organelles following an endosymbiotic event began with descriptions of plastid origins from free-living cyanobacteria by Konstantin Mereschowsky in the early 20th century (see translation by Martin and Kowallik, 1999), which were extended to mitochondria by Ivan Wallin (Wallin, 1927) and later expanded upon and popularized by Lynn Margulis (Sagan, 1967). The endosymbiotic theory was controversial in its infancy but gained acceptance once analysis of mitochondrial DNA (mtDNA) showed an unambiguous phylogenetic connection to proteobacteria (Andersson et al., 1998; Bonen et al., 1977; Gray, 1983; Gray et al., 1998, 2001; Muñoz-Gómez et al., 2017; Roger et al., 2017; Wang and Wu, 2015). Although still incompletely elucidated, current theories for eukaryogenesis propose an endosymbiotic event where an alphaproteobacterium was engulfed by a host cell originating from the Asgardarchaeota superphylum (Spang et al., 2015). This event resulted in a mitochondria-containing last eukaryotic common ancestor (LECA) (Roger et al., 2017). Morphological similarities between mitochondrial cristae structure and intracytoplasmic membranes (ICM) in bacteria, which canonically function to increase membrane surface area for bioenergetic processes akin to their cristae counterparts (Niederman, 2006), have also been proposed as lines of evidence for the prokaryotic origins of mitochondria (Degli Esposti, 2014; Muñoz-Gómez et al., 2017). More recent studies have delineated the alphaproteobacterial origins of the cristae-shaping MICOS complex (Huynen et al., 2016; Muñoz-Gómez et al., 2015a, 2023), establishing an evolutionary connection between modern cristae structures and ICMs.

Determining the prokaryotic origins of cristae-shaping proteins is complemented by research on the origin of mitochondrial lipids, centered around the metabolism of CL (Geiger et al., 2023; Raval et al., 2023). The bacterial origins of CL have putatively been identified using phylogenetics (Luévano-Martínez and Duncan, 2020; Tian et al., 2012), but definitive metabolic comparisons between prokaryotes and eukaryotes are still scarce (Rappocciolo and Stiban, 2019). To this end, this Review will summarize current knowledge on the diversification of mitochondrial-specific lipidomes following eukaryogenesis and will delineate models for how these lipidic features relate to the evolution of high-curvature cristae structures from more primitive ICMs in prokaryotes. We will first address the minimum features required for evolution of cristae – curvature-inducing proteins – but will also present possible relationships between these structures and lipidic contributions as derived from contrasting prokaryotic and mitochondrial membrane compositions. Our comparisons reveal the need for further investigation into alphaproteobacterial lipid metabolism to bridge the gap in knowledge between the proteinaceous and lipidic origins of cristae morphologies.

## Current models of mitochondrial origins

The endosymbiotic theory, which attests that mitochondria evolved from an alphaproteobacterium that was engulfed by a host cell (related to Asgard archaea), has cemented itself as a focal point for understanding the origins of eukaryotic cells (Martin et al., 2015). Although initially controversial, analysis of universally encoded ribosomal RNA genes from mitochondrial DNA (mtDNA) has corroborated the bacterial origins of mitochondria (Bonen et al., 1977; Gray, 2012). In particular, analysis of slow-evolving plant mitochondrial rRNAs led to the identification of the class alphaproteobacteria as the primary mitochondrial ancestor (Gray et al., 1999; Schnare and Gray, 1982; Spencer et al., 1984; Yang et al., 1985). More recent phylogenomic, biochemical and metabolic analyses have led to the widespread acceptance of the theory (Roger et al., 2017; Wang and Wu, 2015). However, several questions remain on the identity of the mitochondrial ancestor, the nature of its symbiotic relationship with archaeal host(s) (Wang and Wu, 2014) and the chronology of the process (Dacks et al., 2016; Shih and Matzke, 2013).

Current investigations into mitochondrial origins focus on elucidating the identity of the alphaproteobacterial ancestor, its metabolism and potential symbiotic interactions with the archaeal host cell. Initial insights came from analysis of the mitochondrial proteome (Gabaldón and Huynen, 2004; Gray, 2015), which includes over 1000 different proteins of which only 15% are involved in energy metabolism and 1% are encoded by mtDNA (Pfanner et al., 2019; Rath et al., 2021). In animals, mtDNA encodes some of the subunits for complexes directly involved in oxidative phosphorylation – complexes I–IV and ATP synthase (complex V). However, some unicellular eukaryotes, such as the Jakobid protists, exhibit much larger mtDNA genomes, encoding several bacterial-like proteins involved in ribosomal protein translation and a bacterial-type RNA polymerase in addition to ETC machinery (Burger et al., 2013; Lang et al., 1997). This evidence, coupled with phylogenomic analysis, suggests that the Jakobid mitochondrial genome represents an intermediate evolutionary stage, retaining a larger subset of the ancestral endosymbiont genes compared to that seen for other eukaryotic lineages, which underwent more extensive gene transfer to the nucleus (Kannan et al., 2014). Phylogenomic studies vying to unearth the closest living mitochondrial relative initially identified the alphaproteobacterial order Rickettsiales, obligate intracellular parasites, as a potential relative of the mitochondrion ancestor (Andersson et al., 1998; Fitzpatrick et al., 2006; Viale and Arakaki, 1994). However, subsequent analyses placed mitochondria as a sister group to other orders of alphaproteobacteria (Abhishek et al., 2011; Thiergart et al., 2012). Thus, the exact phylogenetic relationship between mitochondria and extant alphaproteobacteria has remained a question under active investigation.

The advent of metagenomics, which allows for identification and genetic profiling of microbial organisms in their native environment independent of culturing (Pérez-Cobas et al., 2020), has led to a resurgence of work and debate on the phylogenetic placement of mitochondria (Fan et al., 2020; Martijn et al., 2018). The inclusion of metagenome-assembled genomes (MAGs) collected from marine samples led to a positioning of the mitochondrial ancestor at the root of most alphaproteobacteria (Martijn et al., 2022; Muñoz-Gómez et al., 2022). In parallel, metagenomics has revolutionized our understanding of the origin of the archaeal host cell, placing it within the Asgardarchaeota superphylum (Eme et al., 2023; Spang et al., 2015; Zaremba-Niedzwiedzka et al., 2017). It is likely that alphaproteobacteria co-evolved with Asgard archaea for some time predating the LECA, rather than endosymbiosis occurring as a single saltational event (Roger et al., 2017). Cultivation of Asgard archaea from environmental samples have allowed for generation of co-cultures that have led to analysis of extant Asgard cell biology (Imachi et al., 2020; Rodrigues-Oliveira et al., 2023). Analogous efforts for environmental alphaproteobacteria will be invaluable in better defining the cell biology and metabolic state of the mitochondrial ancestor.

## Generation of membrane curvature by conserved cristae and ICM-shaping proteins

**Fig. 1.**
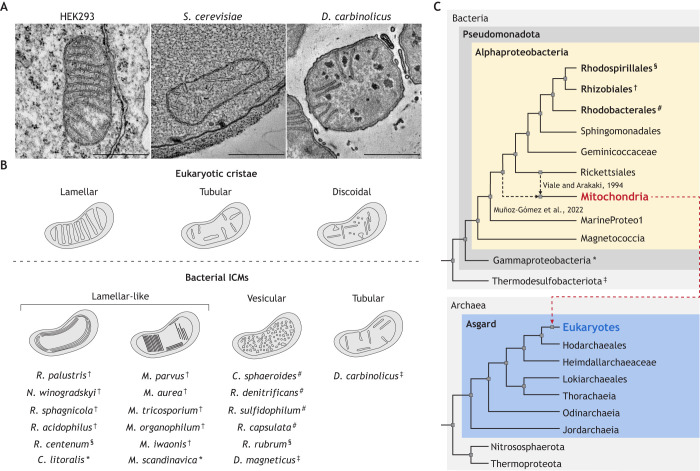
**Bacterial ICMs exhibit internal invaginations that resemble eukaryotic cristae.** (A) Thin-section TEM images showing tubular cristae-like ICM compartments in free-living *Desulfobacterota*, and similar cristae-like ICM tubules in *Desulfovibrio carbinolicus* strains, isolated from the same consortium; images derived from studies presented in McGlynn et al., (2022)*.* The eukaryotic model organism *S. cerevisiae* exhibits tubular cristae structures, whereas the commonly used human embryonic kidney (HEK293) cell line exhibits predominantly lamellar cristae structures. Scale bars: 500 nm. *S. cerevisiae* image derived from samples prepared as in Venkatraman et al. (2023); HEK293 image derived from samples prepared as in Venkatraman and Budin (2024). (B) Eukaryotic mitochondria tend to exhibit lamellar, tubular or discoidal cristae morphology, whereas orders of the class alphaproteobacteria exhibit a range of ICM morphologies. Lamellar-like ICMs, which exhibit parallel cytoplasmic protrusions that do not contact the inner membrane, are commonly observed in nitrogen-fixing and several methanotrophic alphaproteobacteria (Degli Esposti, 2014; Kip et al., 2011; Muñoz-Gómez et al., 2017). Vesicular ICMs are well characterized from TEM analysis of *Cereibacter sphaeroides* and types of magnetotactic alphaproteobacteria (Muñoz-Gómez et al., 2023; Schüler, 2008). (C) Previous phylogenetic results suggested mitochondria as relatives of the alphaproteobacterial order Ricketsialles (Viale and Arakaki, 1994); however, the current phylogenetic consensus places mitochondria as sister to the entire alphaproteobacteria clade (Aouad et al., 2022; Eme et al., 2023; Muñoz-Gómez et al., 2022). This simplified and non-exhaustive cladogram indicates the two domains of life – bacteria and archaea – with proto-mitochondria emerging in early alphaproteobacteria. Eukaryogenesis is marked by the horizontal transfer (red dotted arrow) from Bacteria to Hordarchaeales in Asgard archaea. §, †, #, * and ‡ connect species listed in B to their respective clades in C.

Cristae structures assume various forms across the eukaryotic domain. The two best-characterized types are flat, lamellar cristae traditionally observed in most mammalian tissues, and tubular cristae seen in organisms such as *Saccharomyces cerevisiae* (Harner et al., 2016; Klecker and Westermann, 2021; Pánek et al., 2020; Unger et al., 2017) (Fig. 1A,B). In addition, discoidal cristae, with a mixture of IMM-attached and unattached disk-like structures, have been identified predominantly in protists such as *Trypanosoma* (Kaurov et al., 2018)*.* Reflecting this structural diversity, prokaryotic ICMs exhibit a range of morphologies. In alphaproteobacteria, lamellar-like structures have been observed, with ICMs parallel but unattached to the inner membrane (IM) (Kulichevskaya et al., 2006; Muñoz-Gómez et al., 2017, 2023; Niederman, 2006; Scott et al., 1981; Watson and Mandel, 1971) (Fig. 1B). Similar lamellar-like ridged ICM structures have also been identified in a range of methanotrophic proteobacterium (Degli Esposti, 2014; Dunfield et al., 2010; Kip et al., 2011; Reed et al., 1980). Vesicular ICMs, with little to no membrane attachment, are found in multiple anoxygenic photosynthesizing alphaproteobacteria (of the orders Rhidozobiales, Rhodobacterales and Rhodospirillales) (Iba et al., 1988; Kaufmann et al., 1982) (Fig. 1B,C), as well as in some magnetotactic bacterium that contain organelle-like structures called magnetosomes (Muñoz-Gómez et al., 2017; Schüler, 2008). Recently, cristae-like compartments have been observed outside of the alphaproteobacterial class in sulfate-reducing free-living *Desulfobacterota* and isolated *Desulfovibrio carbinolicus* belonging to the Thermodesulfobacteriota phylum (McGlynn et al., 2022), with lamellar ICM shapes comparable to those of eukaryotic cristae (Fig. 1A). Though apparently dissimilar from mitochondrial cristae, ICMs have been proposed to be linked to cristae, both by the presence of components homologous to MICOS and the aerobic ETC and by their roles in compartmentalization of bioenergetic processes (Muñoz-Gómez et al., 2017). However, further molecular characterizations of bacterial ICMs are required to delineate evolutionary links with cristae morphologies.

**Fig. 2.**
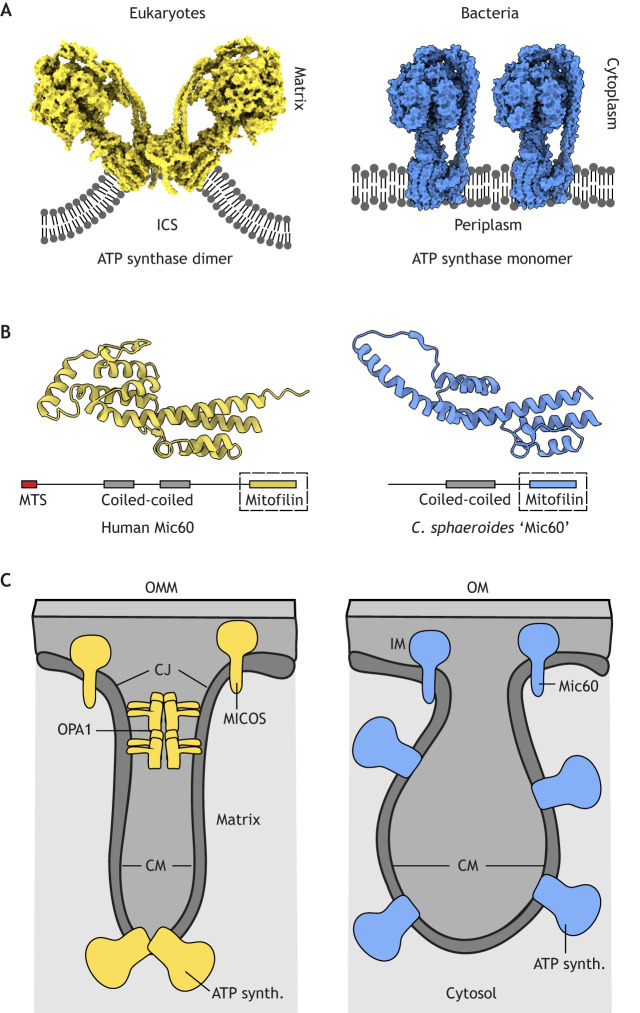
**Proteinaceous features of eukaryotic cristae and prokaryotic ICMs.** (A) Eukaryotic cristae feature rows of ATP synthase dimers (PDB ID: 7AJF) that provide positive curvature at the cristae tips. ATP synthase dimerization subunits are absent in bacteria, which exhibit ATP synthase monomers (PDB ID: 6OQR) lacking membrane bending capabilities compared to the dimeric form (Daum et al., 2013; Paumard et al., 2002). (B) AlphaFold 2 structural depictions of the conserved C-terminal mitofilin domains of human Mic60 (Uniprot ID: Q16891, 562–758) and the putative *Cereibacter sphaeroides* Mic60 homolog (Uniprot ID: Q3J6D4, 270–433), adapted from Benning et al. (2024) preprint. (C) At CJs, OPA1 and MICOS interact to tighten the junction, giving rise to negative curvature. Bacteria lack OPA1, but some alphaproteobacteria like *Cereibacter sphaeroides* retain homologs of Mic60, which could presumably account for some curvature at ICM–IM junctions. We speculate that despite the absence of ATP synthase dimerization, the presence of a Mic60 homolog creates the bulbous appearance of ICMs observed in many alphaproteobacteria, as junctions retain some tightness while curvature at ICM tips is absent. MTS, mitochondrial targeting signal; CM, cristae membrane; OM, outer membrane; IM, inner membrane.

Despite the apparent diversity of cristae and ICM morphologies, high membrane curvature might be a hallmark of these structures and could mediate their specific bioenergetic functions. In mitochondria, positive membrane curvature is generated predominantly by rows of ATP synthase dimers that bend the membrane along cristae tips (Blum et al., 2019), whereas membrane deformation by MICOS produces negative curvature at attached crista junctions (CJs) (Rabl et al., 2009; Tarasenko et al., 2017; Zuccaro et al., 2024 preprint). By contrast, alphaproteobacteria and other prokaryotes lack ATP synthase dimerization subunits (e and g) (Kühlbrandt, 2015, 2019), which suggests that membrane bending by ATP synthase and positively curved cristae tips evolved during eukaryogenesis (Fig. 2A). Genetic deletions of subunits e and g in *S. cerevisiae* result in monomeric ATP synthases and multi-lamellar onion-shaped cristae. These morphologically resemble lamellar prokaryotic ICMs (Klecker and Westermann, 2021; Paumard et al., 2002; Rabl et al., 2009; Venkatraman et al., 2023), although further volumetric electron microscopy (EM) analyses are required to definitively assess these similarities. Monomeric ATP synthases retain the ability to localize into regions of high curvature *in vitro* (Valdivieso González et al., 2023), and the overproduction of subunit b of ATP synthase in *Escherichia coli* (which contain monomeric ATP synthases and do not naturally form ICMs) drives ICM formation (Arechaga et al., 2000; Carranza et al., 2017)*.* Although OPA1 is thought to have become specialized in opisthokonts, a eukaryotic supergroup which includes fungi and animals (Hashimi et al., 2024), homologs of the major MICOS subunit Mic60 have been identified in alphaproteobacteria, suggesting that its presence predates eukaryogenesis (Huynen et al., 2016; Muñoz-Gómez et al., 2015a, 2022, 2023) (Fig. 2B). Several studies have demonstrated comparable functions for Mic60 in alphaproteobacteria and eukaryotic cristae. Genetic deletion of *mic60* in alphaproteobacterial strains reduces growth in conditions that required ICM development, suggesting a conserved functional role for Mic60 (Muñoz-Gómez et al., 2023). Furthermore, alphaproteobacterial Mic60 has been shown to be capable of membrane deformation *in vitro* and, strikingly, its heterologous expression in Mic60-deficient *S. cerevisiae* partially rescued cristae morphology (Tarasenko et al., 2017). These results imply that curvature generation by Mic60 at ICM–IM junctions may have predated the formation of negatively curved CJs in eukaryotes (Rabl et al., 2009).

The interplay between positive and negative curvatures at cristae tips and CJs, respectively, could be the key to understanding the structural transitions between ICMs and cristae during eukaryogenesis. Based on the evidence summarized above, prokaryotic ICMs, particularly those with vesicular topologies, likely exhibit low positive curvature at ICM tips as a result of the reduced membrane bending capabilities and curvature generation of monomeric ATP synthase compared to the dimeric form (Daum et al., 2013; Davies et al., 2012; Paumard et al., 2002). ICMs might still exhibit negative curvature from Mic60 homologs at ICM–IM junctions, which could explain the appearance of bulbous ICM architectures in some alphaproteobacteria (Fig. 2C). However, this argument is complicated by the presence of long-lamellar and tubular type prokaryotic ICMs, such as those in *R. palustris* and *D. carbinolicus*, respectively, which might have curvature properties more comparable to those of mitochondrial cristae. An additional confounding factor is the presence of alternative curvature-generating protein factors in prokaryotic ICMs, such as the light harvesting complexes LH1 and LH2, which have been shown to induce positive curvature on specific photosynthetic ICM invaginations known as chromatophores (Chandler et al., 2008).

During mitochondrial evolution, OPA1-induced membrane curvature could have facilitated the oligomerization of ATP synthase (Quintana-Cabrera et al., 2018) and stabilized CJs by physically interacting with the MICOS complex (Glytsou et al., 2016; Schweppe et al., 2017). However, studies in mammalian cells have shown that CJs can still form in the absence of OPA1 (Barrera et al., 2016), suggesting that negative curvature at CJs might have preceded the incidence of OPA1, and that MICOS might be the primary driver of CJ formation whereas OPA1 is a key regulator of overall cristae shape. Notably absent from this model are roles for curvature-inducing mitochondrial glycerophospholipids – which vary significantly between prokaryotes and mitochondria – in shaping the formation of high-curvature cristae structures.

## Features and evolution of mitochondrial lipid metabolism

Mitochondria exhibit several PLs whose characteristics differ from those found in other organelles. A well-known illustration of this is the presence of CL, which is exclusively synthesized and localized in the IMM. CL is unique among PLs in that it contains four acyl chains linked by two glycerol-phosphate moieties (LeCocq and Ballou, 1964). This distinctive molecular geometry confers biophysical properties that fundamentally influence membrane organization and dynamics within mitochondria. Biophysical studies have demonstrated that membrane curvature affects CL distribution, with CL showing pronounced enrichment in regions of high negative curvature (Beltrán-Heredia et al., 2019). This spatial organization reflects how CL structure enables efficient packing in curved membranes. Recent quantitative analyses reveal that CL exhibits stronger curvature-dependent sorting than other non-bilayer phospholipids like PE (Golla et al., 2024), suggesting a role for CL in stabilizing cristae through preferential accumulation at curved regions.

**Fig. 3.**
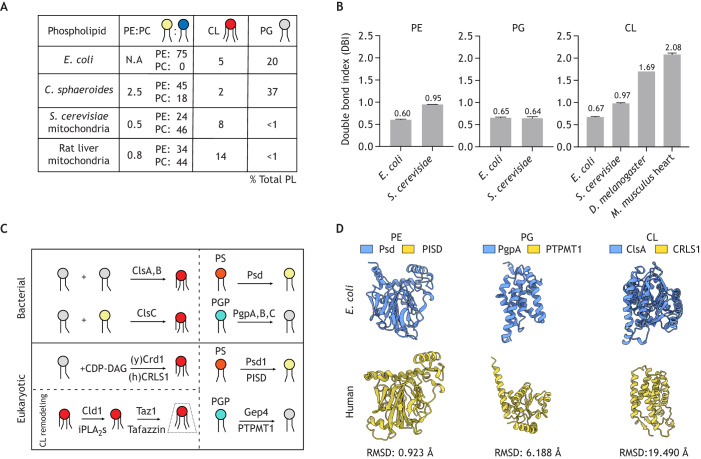
**Lipid biosynthesis demarcates differences between bacterial and mitochondrial membranes.** (A) In *E. coli* and the alphaproteobacterial *Cereibacter sphaeroides*, PE (yellow) and PG (gray) are the major PL constituents and CL (red) is present in low quantities. PC (blue) is absent from *E. coli* but is present in various alphaproteobacterial lipidomes, including in *Cereibacter sphaeroides*, although notably with lower abundance than in mammalian cells. CL levels significantly increase in eukaryotic mitochondria, particularly in mammals, accompanied by major reductions in PE and PG. PC is the most abundant PL in eukaryotic mitochondria. Lipidomics data obtained from Budin et al. (2018), Venkatraman et al. (2023), and Horvath and Daum (2013) for *E. coli* whole cells, and mitochondria isolated from *S. cerevisiae* and rat liver, respectively. (B) Unsaturated fatty acids are selectively driven toward synthesis of PE and CL in mitochondria. In *E. coli*, PE, CL and PG exhibit identical levels of unsaturation, whereas PE and CL are maximally unsaturated in *S. cerevisiae* mitochondria, as denoted by a double bond index (DBI; which describes the mean unsaturation per acyl chain) close to 1. In *Drosophila melanogaster* and *Mus musculus* heart, CL exhibits nearly homogenous, tetra-linoleic (C18:2)_4_ acyl chains (DBI close to 2). Lipidomics data obtained from Budin et al. (2018), Venkatraman et al. (2023) and Zhu et al. (2021) for *E. coli* (*n*=3), *S. cerevisiae* (*n*=3) and *M. musculus* (*n*=5), respectively. *D. melanogaster* (*n*=1) lipidomics were completed on whole flies. Error bars indicate s.d. (C) PL biosynthesis in bacteria versus mitochondria primarily differs in biosynthesis of CL. CL is synthesized by two pathways in *E. coli*, both catalyzed by a phospholipase D (PLD)-type CL synthase – from a phosphatidyl transfer either between two PG molecules, via ClsA or ClsB, or from PE to PG, via ClsC. In contrast, in a process restricted to eukaryotes, CL is synthesized from a phosphatidyl transfer from CDP-DAG to the free hydroxyl of the PG to form *de novo* CL, which is then remodeled in two-steps by a deacylation (by Cld1 in yeast and multiple iPLA_2_ enzymes in mammals) and transacylation (by Taz1 in yeast and tafazzin in mammals) to form homogeneously unsaturated CL. The decarboxylation of PS (orange) into PE is catalyzed by enzymes conserved between bacteria and mitochondria: PISD in humans and Psd in *E. coli.* Similarly, PGP (cyan) is dephosphorylated to form PG in both mitochondria and bacteria, but the phosphatases that catalyze this reaction differ. (D) Structural similarities and divergences based on calculated root mean square deviations (RMSD) between phospholipid metabolic enzymes from mitochondria and bacteria. RMSD values are calculated using atomic distances between superimposed structures, with lower RMSD values indicating higher degrees of structural similarity. AlphaFold 2 structural depictions are shown for human PISD (Uniprot ID: Q9UG56), *E. coli* Psd (Uniprot ID: P0A8K1), human PG synthase (PTPMT1, Uniprot ID: Q8WUK0) and one of three PGP phosphatases of *E.coil* (PgpA, Uniprot ID: P18200). CL synthases carry out different reactions in bacteria and mitochondria and their enzymes are not homologous. Shown are ClsA from *E. coli* (Uniprot ID: P0A6H8) and human CRLS1 (Uniprot ID: Q9UJA2).

The abundance of CL in eukaryotes varies significantly across organisms and even within tissues. Studies from *S. cerevisiae* have reported that CL constitutes between 8–10% of total mitochondrial PLs (Baile et al., 2014; Basu Ball et al., 2018b; Claypool et al., 2011; Venkatraman et al., 2023), whereas analyses in rat liver mitochondria report levels closer to 15% (Daum, 1985; Horvath and Daum, 2013) (Fig. 3A). By contrast, CL seems to be less prevalent in bacterial membranes, constituting ∼5% of total PLs in *E. coli* (Fig. 3A) (Budin et al., 2018; Rowlett et al., 2017)*.* CL is also found in many alphaproteobacteria (Sohlenkamp and Geiger, 2016). For instance, CL has been detected in *Cereibacter sphaeroides*, *Rhizobium tropici* and *Agrobacterium tumefaciens* at abundances between 2% and 5% of total PLs (Czolkoss et al., 2016; Lin et al., 2015; Vences-Guzmán et al., 2011), with one study reporting above 10% CL in *Rhizobium meliloti* (Thompson et al., 1983). Some non-alphaproteobacterial genera also display very high CL abundances, with it being above 10% of total PLs in *Legionella pneumophila* (Conover et al., 2008) and 30% in *Xanthomonas campestris* (Moser et al., 2014); however, it remains unclear how these increased CL levels contribute to membrane biogenesis and function.

CL fulfills some similar functions in prokaryotes and eukaryotes; it binds to and stabilizes ETC complexes in both eukaryotes (Mehdipour and Hummer, 2016; Mühleip et al., 2019; Mühleip et al., 2023; Pfeiffer et al., 2003) and prokaryotes (Arias-Cartin et al., 2012). Work from our group in *S. cerevisiae* has demonstrated the essentiality of CL in shaping cristae structures under conditions of reduced assembly of ATP synthase dimers (Venkatraman and Budin, 2024). This suggests that CL contributes independently to cristae morphology, potentially by relaxing stress that arises from large negative-curvature regions (Beltrán-Heredia et al., 2019; Venkatraman et al., 2023). Intriguingly, modulation of local pH is sufficient to produce cristae-like membrane invaginations in giant unilamellar vesicles in a CL-dependent manner. This phenomenon likely results from a change in CL packing in the monolayer when exposed to more protons (Khalifat et al., 2008, 2011). In line with these observations, several studies have also shown the curvature-based sorting of CL in bacteria (Kawai et al., 2004; Mileykovskaya and Dowhan, 2000; Renner and Weibel, 2011). In parallel, recent investigations have revealed a synergistic dependency between CL and ATP synthase for the formation of ICMs in *E. coli*, in which ICMs do not naturally form, but can be induced upon ATP synthase subunit b overexpression (Carranza et al., 2017). In that study, the loss of CL led to the production of onion-like multi-lamellar ICM structures at the cell periphery rather than long lamellar ICMs throughout the cytoplasm. Taken together, the biophysical contributions of CL and its interactions with ETC enzymes and ATP synthase imply that this lipid has played a key role in the development of cristae-like inner membrane shapes during eukaryogenesis.

Although the abundance and functions of CL likely increased during mitochondrial evolution, PE and phosphatidylglycerol (PG) levels decreased, highlighting key distinctions between bacterial and mitochondrial lipidomes (Fig. 3A). For example, in mitochondria, PG is synthesized strictly as a substrate for CL synthesis and does not itself accumulate to significant levels (Osman et al., 2011). Furthermore, although absent in many bacteria including *E. coli*, phosphatidylcholine (PC) is the most abundant mitochondrial PL, comprising over 40% of total mitochondrial PLs (Fig. 3A) (Daum, 1985; Geiger et al., 2013). Interestingly, although *E. coli* lacks detectable PC, alphaproteobacteria, such as *Cereibacter sphaeroides* and *Methylobacterium organophilum*, exhibit PC levels that are close to 20% of total PLs (Benning et al., 1993; Donohue et al., 1982; Patt and Hanson, 1978) (Fig. 3A). PC synthesis is a common reaction in alphaproteobacteria, as exemplified in *Sinorhizobium meliloti* and other relatives (de Rudder et al., 1999; Martínez-Morales et al., 2003). However, in contrast to what occurs in eukaryotes, bacterial PC synthesis couples free choline to cytidine diphosphate (CDP)-diacyl glycerol (DAG), instead of CDP-choline as in the eukaryotic Kennedy pathway. The PE:PC ratio in mitochondria is also much lower than the ratios measured in PC-producing alphaproteobacteria. Strains of *Cereibacter sphaeroides* exhibit high (>2) PE:PC ratios (Donohue et al., 1982), but in mammalian mitochondria the ratio is about 0.8 (Horvath and Daum, 2013) (Fig. 3A). The IMM has nearly twice the PE:PC ratio of the OMM (1.15 versus 0.64, respectively) (Hovius et al., 1990), which likely supports a requirement of PE for bioenergetic activity and cristae architecture (Basu Ball et al., 2018a; Calzada et al., 2016; van der Veen et al., 2017). In addition to these differences in PE and PC abundances, PG is a major PL (≈35%) of *Cereibacter sphaeroides* and other alphaproteobacteria (Fig. 3A) (Benning et al., 1993; Donohue et al., 1982; Rowlett et al., 2017) but shows only trace detection in mitochondria. This alteration could potentially be attributed to increased demand for CL production within mitochondria, which has relegated PG to a transient intermediate in CL synthesis (Osman et al., 2011). It has been suggested that PG can compensate for several functions of CL when it accumulates upon ablation of CL synthase in *S. cerevisiae* (Jiang et al., 2000), potentially supporting this ancestral role.

In addition to altered head group chemistries, bacterial and mitochondrial membranes differ in their fatty acid compositions. Mitochondria are enriched in unsaturated PLs and exhibit low levels of saturated PLs, even upon genetic inhibition of desaturases (Venkatraman et al., 2023). Increasing acyl chain unsaturation is correlated with increased spontaneous curvature in a variety of PLs (Dymond, 2021; Venkatraman et al., 2024). The unsaturated fatty acid pool within mitochondria is shunted towards nonbilayer-preferring PE and CL lipid species, which, in their fully unsaturated forms, are thought to provide the necessary curvature for cristae formation (Basu Ball et al., 2018a). PL acyl chain compositions are described by their double bond index (DBI), the mean number of unsaturations per acyl chain (Vornanen et al., 1999; Winnikoff et al., 2021). In *S. cerevisiae* mitochondria, which cannot synthesize polyunsaturated fatty acids, the predominant CL and PE species are monounsaturated on each acyl chain (with a DBI close to 1), whereas the CL precursor, PG, predominantly contains one monounsaturated and one saturated acyl chain (DBI≈0.6) (Fig. 3B). *E. coli* membranes display increased levels of saturation, and PG, PE and CL species exhibit nearly identical acyl chain profiles, suggesting a lack of PL specificity in unsaturated fatty acyl flux in bacterial membranes. In eukaryotic mitochondria, selective unsaturation of PE might be derived from preferential import of unsaturated phosphatidylserine (PS) at ER–mitochondria contact sites for PE synthesis by phosphatidylserine decarboxylase (PSD, called Psd1 in yeast and PISD in mammals) (Heikinheimo and Somerharju, 1998, 2002; Renne et al., 2022). In eukaryotes, the high level of unsaturation in CL is attributed to a two-step remodeling pathway in which CL is first deacylated by a phospholipase A_2_ (Cld1 in yeast, multiple PLA_2_ enzymes in mammals) before a transacylase transfers unsaturated fatty acyl chains from PC or PE to CL in multiple steps to attain homogeneously unsaturated CL (Abe et al., 2016; Schlame et al., 2012, 2017). In mammals, which can synthesize polyunsaturated fatty acids, CL acyl chains are typically linoleic (18:2, a C18 chain with two unsaturations), and thus have a DBI close to 2 (Oemer et al., 2018, 2020; Schlame et al., 2005) (Fig. 3B). The enzymes involved in the two-step remodeling of CL (deacylation-reacylation) are absent in alphaproteobacteria and other prokaryotes but are also not ubiquitous in eukaryotes, as certain unicellular protists and fungi lack homologs of both PLA_2_ deacylation enzymes and the best-characterized CL transacylase, tafazzin (Taz1 in yeast) (Tian et al., 2012). Although studies reporting CL acyl chain compositions from alphaproteobacteria are limited, a study in *Agrobacterium* has shown that CL exhibits slightly more unsaturated fatty acyl species than the total fatty acid pool (Czolkoss et al., 2016), which might indicate alternative remodeling machinery in alphaproteobacteria. Further studies are thus necessary to elucidate the origins of CL remodeling and their comparative roles in alphaproteobacteria and eukaryotes.

The relationship between oxygen utilization during eukaryogenesis and cristae shape might be linked by adaptations in unsaturation levels. According to some models, the incidence of early protomitochondria occurred after a surge in atmospheric oxygen 2.4 billion years ago (de Duve, 2007; Hall, 1973; Mills et al., 2022), albeit to levels much lower than those of today. This event likely coincided with emergence of a capacity for aerobic respiration in cells and concurrently the formation of invaginated cristae-like membranes. The direct interplay between cristae biogenesis and oxygen consumption is not fully understood but is thought to be driven by ETC-based changes (Fuhrmann and Brüne, 2017). Lipid unsaturation is also a key regulator of cristae structure and electron carrier diffusion in the ETC (Budin et al., 2018). The enzymes that desaturate acyl chains in eukaryotes, such as yeast Ole1 or mammalian SCD1, are oxygen dependent (Kwast et al., 1999; Vasconcelles et al., 2001), and their inhibition in hypoxic environments increases lipid saturation levels (Ackerman et al., 2018; Kamphorst et al., 2013; Venkatraman et al., 2023). By contrast, some bacteria such as *E. coli* produce unsaturated fatty acids anaerobically during *de novo* fatty acid biosynthesis (Cronan, 2024; Cronan and Thomas, 2009) but are more limited in their compositional diversity. Oxygen limitation in *S. cerevisiae* cells, which are specialized for microaerobic fermentation, increases total PL saturation but also drives a dependency on CL synthesis for maintenance of cristae structure (Venkatraman and Budin, 2024). PL acyl chain composition in this system better mimics the more saturated lipidomes of bacteria, and we posit that the requirement of CL for cristae formation under these conditions might imply an early role for CL in shaping cristae or ICMs under reduced oxygenation, which could be tested by genetic deletion of CL synthesis in ICM-forming alphaproteobacteria. The subsequent proliferation of highly unsaturated mitochondrial membranes could have been dictated by the increase in oxygenation of the earth over the past billion years.

## The phylogeny of phospholipid metabolism in mitochondria

Mitochondria harbor machinery to synthesize a portion of the PLs that make up their membranes. Biosynthesis for PE, PG and CL all occur in the IMM. Although a portion of cellular PE is produced in the ER (Kennedy and Weiss, 1956), mitochondrial PE is made from imported PS, which is decarboxylated by Psd1 or PISD (Fig. 3C) (Acoba et al., 2020; Horvath et al., 2012). Analogously in *E. coli*, a PSD (Psd) conducts a reaction identical to that in eukaryotes to produce PE, but PE can also be derived from CDP-ethanolamine in an alternative pathway (Sohlenkamp and Geiger, 2016). Both bacterial and eukaryotic PSDs are initially synthesized as inactive proenzymes (Schuiki and Daum, 2009). They exhibit sequence and structural homology (Fig. 3D), exemplified by their conserved C-terminal LGST motif, which marks the autocatalytic cleavage site necessary for PSD enzymatic activity (Cho et al., 2021; Schuiki and Daum, 2009; Voelker, 1997).

In mitochondria, PG is synthesized as a precursor to CL, both of which are derived from CDP-DAG that originates from ER-supplied phosphatidic acid (PA). PG synthesis in both mitochondria and prokaryotes occurs via a PG phosphate intermediate generated from CDP-DAG (Chang et al., 1998; Gopalakrishnan et al., 1986). In bacterial cells, three phosphatases (PgpA, PgpB and PgpC) catalyze the dephosphorylation of phosphatidylglycerol phosphatase (PGP) to form PG (Lu et al., 2011), whereas the phosphatases PTPMT1 and Gep4 conduct identical reactions in mammals and yeast, respectively (Osman et al., 2010; Zhang et al., 2011) (Fig. 3C). Although bacterial PgpA and PgpB enzymes do not show structural or sequence homology with Gep4 or PTPMT1 (Fig. 3D), mitochondrially targeted PgpA has been shown to functionally compensate for Gep4 deficiency in *S. cerevisiae*, indicating functional conservation of PG dephosphorylation between prokaryotes and mitochondria (Osman et al., 2010). In bacteria, PG receives a phosphatidyl group from another PG molecule to form CL (Schlame, 2008); this reaction is catalyzed by a phospholipase D (PLD) enzyme (ClsA or ClsB) (Hirschberg and Kennedy, 1972). More recently, another member of the PLD family, ClsC, has been shown to catalyze the transfer of a phosphatidyl group from PE to PG to form CL (Tan et al., 2012). In contrast, in eukaryotes, CL synthase (CRLS1 in mammals, Crd1 in yeast) transfers a phosphatidyl group from CDP-DAG to PG to form CL, a reaction that occurs in the matrix-facing side of the IMM (Fig. 3C) (Hostetler et al., 1971, 1972; Schlame and Haldar, 1993). Reflecting the different enzymatic reactions they conduct, eukaryotic and bacterial CL synthases are not homologous (Fig. 3D).

Phylogenetic analyses have revealed the presence of bacterial-type CL synthases in several eukaryotic groups, including Alveolata and Kinetoplastids (Serricchio and Bütikofer, 2012; Tian et al., 2012). Interestingly, although most prokaryotes exhibit PLD-type CL synthases, some alphaproteobacteria bear both bacterial and eukaryotic-type CL synthesis machinery, suggesting that proto-mitochondria might also have borne both CL synthesis mechanisms (Geiger et al., 2023). Additional biochemical, lipidomic and phylogenetic analyses are required to further link CL synthesis to the metabolic state of the proto-mitochondrion and to better understand how mitochondrial and bacterial CL pathways diverged.

## Conclusions and perspectives

The advent of extensive metagenomic, phylogenetic and molecular biology tools have already advanced our understanding of mitochondrial origins. However, several questions remain regarding the energetics of the proto-mitochondrion, its interactions with the host cell and the chronology of the endosymbiotic event. Inferring the characteristics of the specific alphaproteobacterial ancestor of mitochondria using phylogenetic tools has been central in efforts to answer these questions, but substantial debate remains (Brindefalk et al., 2011; Degli Esposti et al., 2014; Muñoz-Gómez et al., 2022; Rodríguez-Ezpeleta and Embley, 2012; Roger et al., 2017). Recent discoveries regarding the alphaproteobacterial origins of Mic60, the major subunit of the cristae-shaping MICOS complex (Huynen et al., 2016; Muñoz-Gómez et al., 2015a,b, 2022, 2023) and the identification of cristae-like ICM invaginations in bacteria (Degli Esposti, 2014; Muñoz-Gómez et al., 2017) have reinvigorated interest in cristae evolution and its pre-endosymbiotic origins. Coupled with phylogenetic analyses, a systematic study of cristae-like structures in candidate alphaproteobacteria and their relationship to respiratory function could be invaluable in identifying the metabolic state of the proto-mitochondrion. Similarly, discerning the molecular determinants of ICM formation, as have been reported for mitochondrial cristae (Cogliati et al., 2016; Pánek et al., 2020), would also provide a framework for understanding how eukaryotes became specialized for aerobic respiration. To this end, the application of high-resolution 3D imaging techniques, such as cryo-electron tomography and focused ion beam scanning electron microscopy (FIB-SEM), to ICM structures will further elucidate their geometric features. These approaches will be needed to understand specific types of ICM topologies (i.e, lamellar or tubular) and their roles in ATP generation, as is now routine for analyses of mitochondrial cristae (Barad et al., 2023; Bílý et al., 2021; Garcia et al., 2019, 2023; Lee et al., 2020; Mendelsohn et al., 2022; Stephan et al., 2020; Suga et al., 2023; Venkatraman et al., 2023).

The identification and characterization of conserved mitochondrial proteins originating from alphaproteobacteria has shed light on the protein repertoire of the proto-mitochondrion, which might provide insight into its energetic state (Andersson et al., 1998; Gabaldón and Huynen, 2004; Gray, 2015). However, these comparisons are complicated by dilution of the phylogenetic signal between bacterial and mitochondrial protein homologs and the differential loss of ancestral alphaproteobacterial genes from mitochondrial genomes, contributing to the small number of conserved proteins encoded in both genomes (Geiger et al., 2023; Kurland and Andersson, 2000; Nagies et al., 2020; Raval et al., 2023). An alternative area of interest comes from comparative analyses of mitochondrial and alphaproteobacterial lipidomes, as we have reviewed here. The determination of lipidic features in the proto-mitochondrion could turn out to be pivotal in depicting its energetic state. In this regard, particular attention should be directed toward the elucidation of evolutionary history of the complete biosynthetic pathways leading to PLs such as PE and CL, which provide indispensable functions in the mitochondria as well as in many alphaproteobacteria. Although phylogenetic analyses have demonstrated the alphaproteobacterial origins of CL synthesis enzymes (Geiger et al., 2023; Tian et al., 2012), further studies could reveal links between membrane lipid composition and the presence of aerobic traits, ICM formation or even the acquisition of more diverse organelles. Although the lipidomes of mitochondria (from yeast and mammals) and *E. coli* are well characterized and provide initial insights into lipidic adaptations during eukaryogenesis, more extensive lipidomic analyses from diverse alphaproteobacteria are required to explain the transition. In a complementary manner, similar investigations are needed in Asgard archaea to elucidate how distinct lipid metabolisms merged within a single cell while maintaining compartments with distinct lipid compositions and architecture over time. By testing links between lipidic adaptation, phylogenetics delineating aerobic or anaerobic traits and ICM formation in alphaproteobacteria, models for mitochondrial origins could be further developed.
